# Fever, convulsions, and rash: a case report of neonatal lupus erythematosus with macrophage activation syndrome

**DOI:** 10.3389/fped.2024.1381493

**Published:** 2024-08-20

**Authors:** Jing Yu, Guohui Xue, Xiaohui Liu, Chunlei Zhan, Jinshi Huang

**Affiliations:** ^1^ Department of Intensive Care, Jiangxi Children’s Hospital, Nanchang, Jiangxi, China; ^2^ Department of Clinical Laboratory, Jiujiang City Key Laboratory of Cell Therapy, Jiujiang NO.1 People's Hospital, Jiujiang, Jiangxi, China; ^3^ Department of Rheumatology and Immunology, Jiangxi Children’s Hospital, Nanchang, Jiangxi, China; ^4^ Department of Gastroenterology, Jiangxi Children’s Hospital, Nanchang, Jiangxi, China; ^5^ Department of Neonatal Surgery, Jiangxi Provincial Children’s Hospital, Nanchang, Jiangxi, China; ^6^ Department of Neonatal Surgery, Beijing Children’s Hospital Affiliated to Capital Medical University, Beijing, China

**Keywords:** neonatal lupus, fever, rash, macrophage activation syndrome, treatment

## Abstract

Neonatal lupus erythematosus (NLE) is a rare acquired autoimmune disease associated with the entry of maternal antibodies into the fetal circulation via the placenta during pregnancy. Macrophage activation syndrome (MAS) is a severe hyperinflammatory disease. Herein, we present a case of NLE with MAS accompanied by fever, convulsions, and rash. High-dose gamma globulin and non-shock doses of steroids can be used as a first-line treatment for NLE with MAS. Fever can be a clinical manifestation of NLE, especially cutaneous lupus. Rash recession could be used to judge whether the disease is effectively controlled by treatment.

## 1 Introduction

Neonatal lupus erythematosus (NLE) is a rare acquired autoimmune disease associated with the entry of maternal anti-Ro/SSA and anti-La/SSB antibodies into fetal circulation via the placenta (1, 2). Mothers of infants with this condition most commonly have systemic lupus erythematosus, Sjogren's syndrome, other connective tissue diseases, or both, or no clinical symptoms at delivery (3, 4). Macrophage activation syndrome (MAS) is a severe and potentially life-threatening hyperinflammatory disease caused by cytokine storms triggered by excessive activation of T lymphocytes and macrophages (5) and is considered a secondary form of hemophagocytic lymphohistiocytosis (HLH) (6), while rheumatic disease-associated HLH is referred to as MAS (7). Cases of MAS concurrent with NLE have only rarely been reported.

## 2 Case presentation

**Figure 1:**
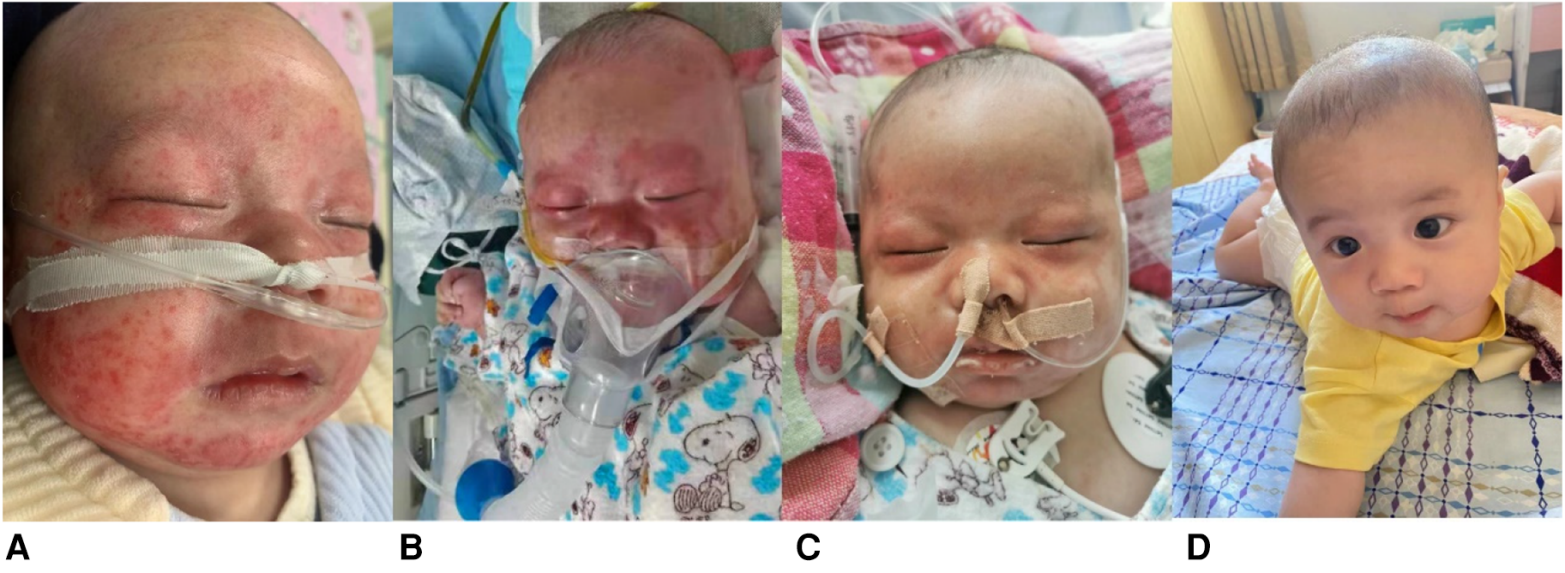
Images demonstrating rash and relief. **(A)** erythematous rash confluent macules over face (Day 7 of illness); **(B)** erythematous macules around periorbital areas (Day 12 of illness); **(C)**, rash over face, especially periorbital areas, partially resolved (Day 16 of illness); **(D)** resolution of rash by four-month follow-up.

Herein, we describe the case of a 2-month 19-day-old male infant who presented to our hospital with a rash lasting 7 days, fever lasting 5 days, intermittent myoclonus lasting 3 days, and one convulsion. His rash was distributed on the face (Figure 1A), and the fever fluctuated between 38.5°C and 39.0°C despite conventional antibiotic treatment (cefotaxime sodium for 3 days, meropenem for 4 days). Myoclonus occurred mainly during fever. The convulsion episode manifested as closed teeth, foaming at the mouth, and tonic exertion of the limbs, with no significant correlation with temperature.

The infant had been delivered at 37 weeks of gestational age, with a birth weight of 3,200 g and a one-minute Apgar score of 10. No abnormal findings were observed during pregnancy or the perinatal or neonatal periods. Maternal antenatal serological testing was negative for rubella, cytomegalovirus, herpes virus, and *Toxoplasma gondii* immunoglobulin (Ig) M. Serological examinations were negative for human immunodeficiency virus, hepatitis B virus, and syphilis. The child's mother denied having any autoimmune or inherited metabolic diseases. Except for the rash, the results of the physical examination were unremarkable.

After admission, the rash gradually spread to the chest, abdomen, soles and around the orbit (Figure 1B). Shortness of breath and recurrence of convulsions with altered consciousness status were also observed. Initial-stage convulsions were classified as focal seizures, manifesting as frequent blinking with corner-of-mouth twitching, which subsequently transformed into paroxysmal increased muscle tone in the limbs. Physical examination revealed that the liver was located 3 centimeters below the ribs and the spleen was located 2 centimeters below the ribs.

Owing to the child's recurrent fever with convulsions, we performed a series of laboratory tests, including routine cerebrospinal fluid tests, biochemistry tests, body fluid and blood cultures, and metagenomic sequencing. The cerebrospinal fluid routine showed a white blood cell count of 0*10^6 ^/L (reference range: 0–15*10^6 ^/L); glucose level, 2.69 (reference range: 2.8–4.5 mmol/L); and protein levels of 1,153 (reference range: 120–600 mg/L). Cerebrospinal fluid IgM tests for antibodies (including mycoplasma, chlamydia, and syncytial viruses) were negative. A cerebrospinal fluid culture was negative for bacterial or fungi. Cranial CT examination showed no significant abnormalities. In conclusion, no positive results were identified to support a central nervous system infection. Peripheral blood IgM tests for viral antibodies (including Epstein-Barr virus, adenovirus, etc.) and, peripheral blood antibodies against atypical pathogens (including mycoplasma and chlamydia) were negative. The Epstein-Barr virus deoxyribonucleic acid titer test was in the normal range. Body fluids culture (sputum and urine), blood cultures and blood metagenomic next-generation sequencing were negative.

**Figure 2:**
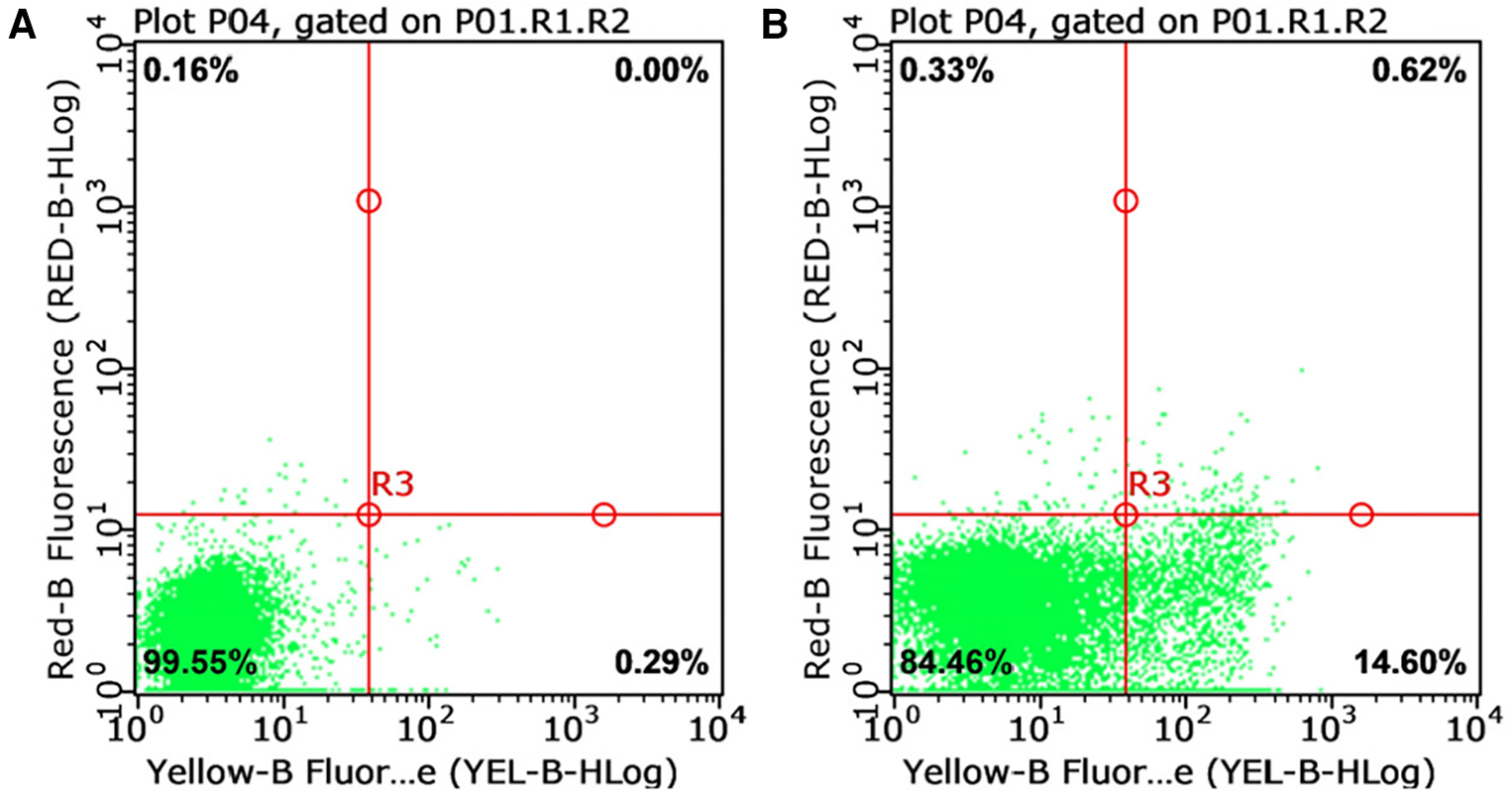
Natural killer cell activity test (reference range: greater than 15.11%). **(A)** Natural apoptosis background of single target cells; **(B)** The killing ratio of natural killer cells to target cells in the submitted samples.

**Figure 3:**
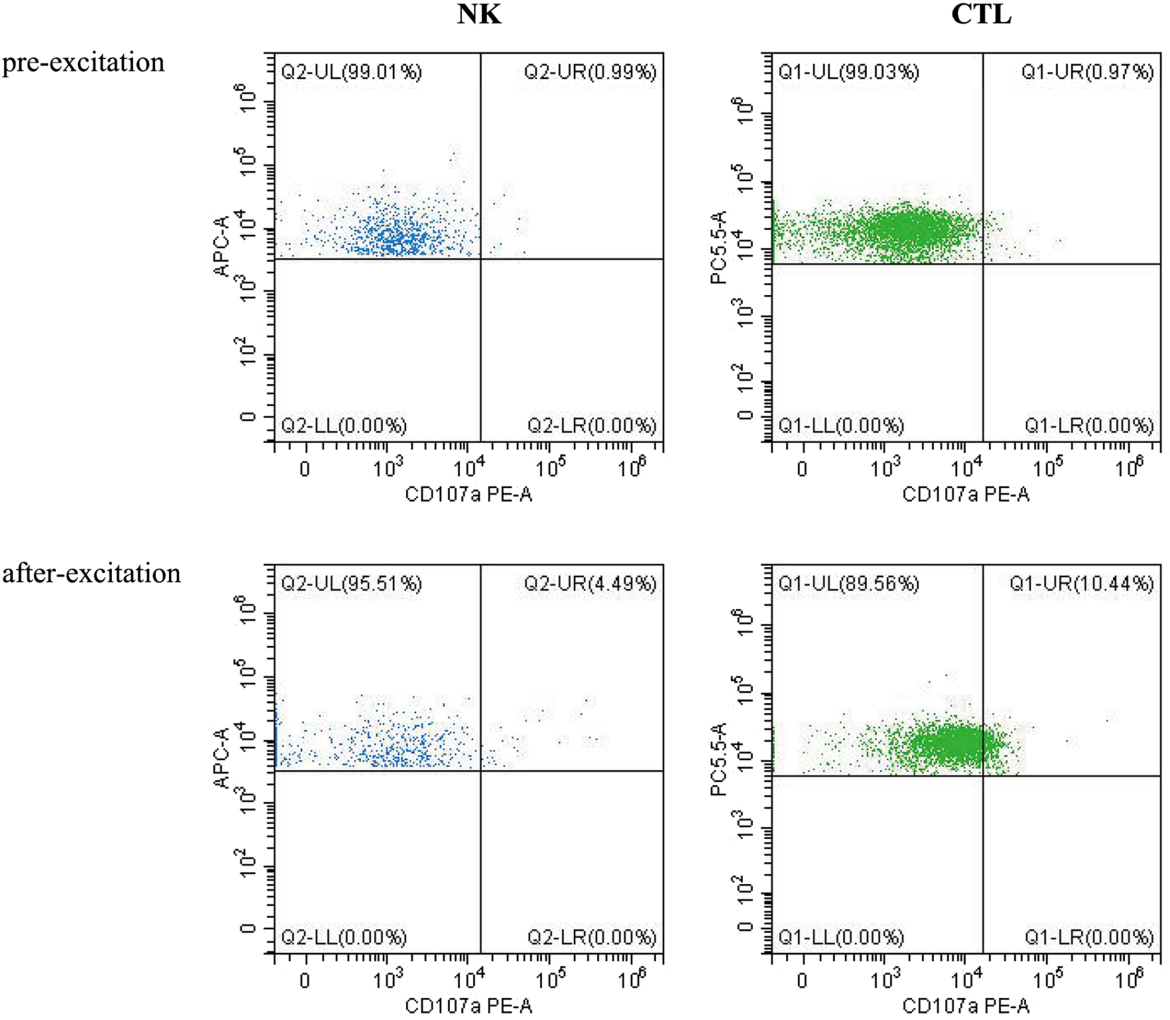
Cd107a excitation assay.

**Figure 4:**
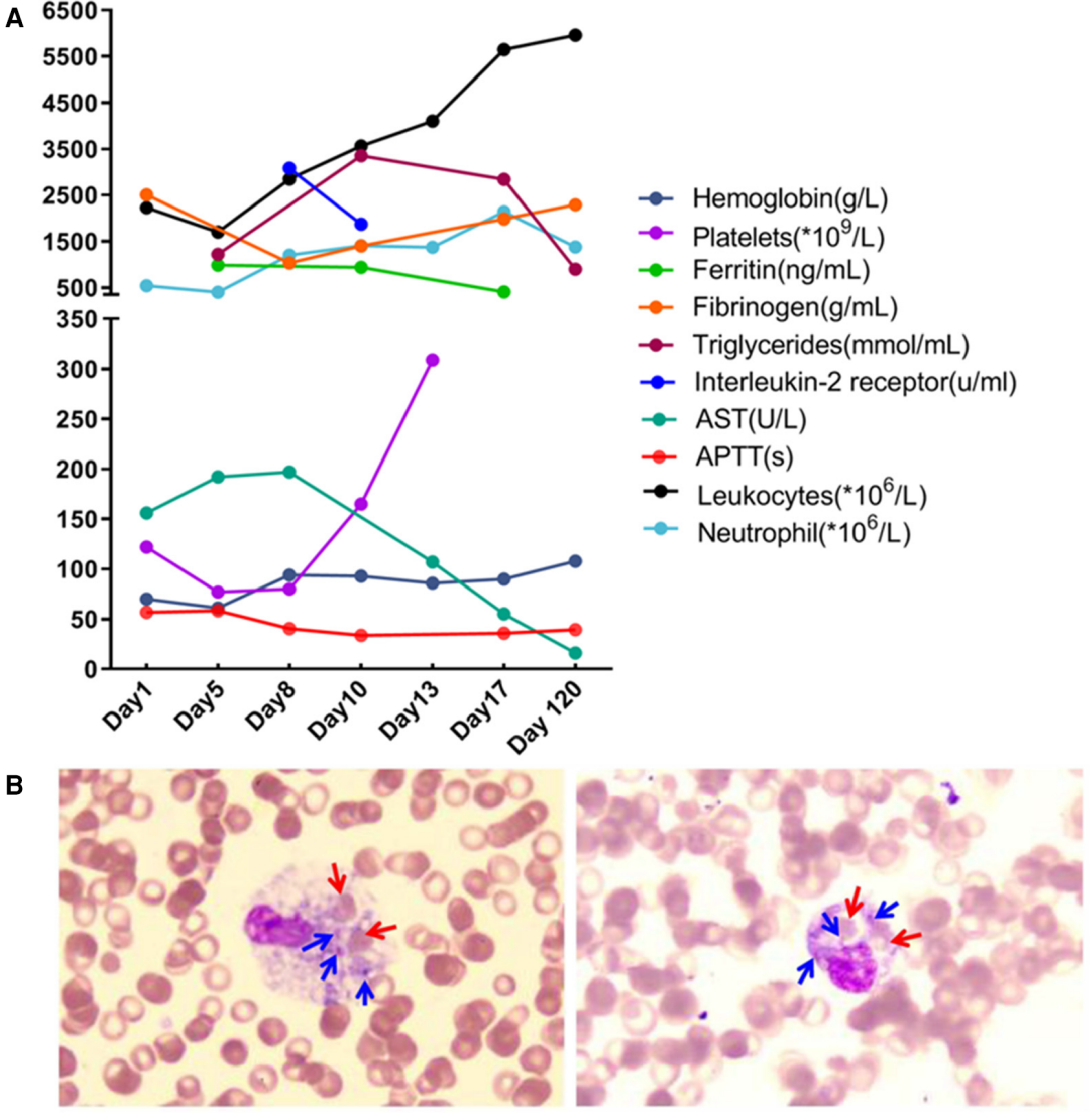
Dynamics of laboratory indices during treatment and follow-up **(A)** and phagocytosis by macrophages in the bone marrow **(B)** Red arrows point to phagocytosed red blood cells; blue arrows point to phagocytosed platelets.

Laboratory investigation revealed a white blood cell count of 1.70 × 10^9 ^/L (reference range: 5.6–14.5*10^9^ /L); hemoglobin level, 66 g/L (reference range: 99–196 g/L); platelet count, 77 × 10^9 ^/L (reference range: 203–653*10^9 ^/L); neutrophil cell count, 0.4 × 10^9 ^/L (reference range: 0.8–6.1*10^9 ^/L); aspartate aminotransferase, 192 U/L (reference range: 21–80 U/L); albumin, 21 g/L (reference range: 35–50 g/L); lactate dehydrogenase, 1,530 U/L (reference range: 100–240 U/L); fibrinogen level, 0.6 g/L (reference range: 1.5–3.76 g/L); activated partial thromboplastin time, 58.3s (reference range: 25.1–40.7 s); ferritin level, 983 ng/ml (reference range: 144–600 ng/ml); triglycerides level, 3.35 mmol/L (reference range: 0.32–0.99 mmol/L); a soluble interleukin-2 receptors level of 3,081 u/ml (reference range: 223–710 u/ml); Flow assay showed natural killer (NK) cells activity at the low limit of the normal range (Figure 2), CD107a excitation assay showed defective degranulation of NK cells, and degranulation of cytotoxic T-cell (CTL) was normal (Figure 3). Initial bone marrow aspiration (3 days after admission) revealed no abnormalities; however, repeat bone marrow aspiration (day 10 after admission) showed hemophagocytosis (Figure 4). The aforementioned laboratory data were consistent with diagnostic criteria for both MAS and 2,004-HLH. To clarify whether MAS/HLH in this case was associated with gene mutations, we performed whole-exome gene sequencing. We identified no mutations in primary HLH (f-HLH)-related genes (*PRF1*, *UNC13D*, *STX11*, *STXBP2*, *SH2D1A*, *XIAP*, *LYST*, *RAB27A*, *AP3B1*, *BIRC4*, *ITK*, *CD27*, or *MAGT1)*, and no secondary HLH-related mutations in P1, *MUNC13–14*, *STXBP2*, or *SYNX11*.

The serum antinuclear antibody profiles of both the child and mother were strongly positive for antibodies against Ro (SS-A) and La (SS-B), with antinuclear antibodies (nuclear granular type) identified at a ratio of 1:320 (normal range, less than 1:100). The mother's diagnosis of Sjorgen's syndrome was confirmed at a general hospital. Based on this information and the skin lesions, especially the periorbital “owl's eye” facial rash, a diagnosis of NLE was presumed.

Other abnormal findings included ultrasounds showing a small amount of bilateral pleural and abdominal effusions and hepatosplenomegaly. A 3 h video electroencephalography (EEG) scan showed slow background activity with no epileptiform discharges. Craniocerebral magnetic resonance imaging (MRI) and magnetic resonance vein angiography revealed multiple diffusion-weighted imaging high-signal shadows in the white matter of the cerebral hemispheres bilaterally and in the head of the corpus callosum geniculate, internal capsule, and caudate nucleus, suggesting cellular edema. No conduction blocks were observed on the child's electrocardiograms.

At the initial stage of admission, we considered central nervous system infection and gave meropenem combined with acyclovir for anti-infection, mannitol to reduce intracranial pressure, phenobarbital to control convulsions, and methylprednisolone for anti-inflammation (1 mg/kg/dose at 12 h intervals). As the child presented with anemia, coagulopathy and hypoproteinemia, we administered a transfusion of erythrocyte suspension, fresh frozen plasma and human albumin but there was no relief in the child's symptoms. After considering MAS as a possible diagnosis, we treated the patient with a high-dose of immunoglobulin (1 g/kg/time/day for 2 days by intravenous infusion) and methylprednisolone (3 mg/kg/time, intravenous drip twice a day, reduced to 1 mg/kg/time after 7 days), thereafter, and the child's fever subsided, his convulsions ceased, and pericardial and pleural effusions were absorbed. The facial rash partially resolved with only ordinary moisturizing skin cream (Figure 1C), and his EEG normalized. Follow-up after four months demonstrated that the rash had completely resolved (Figure 1D); cranial MRI showed bilateral widening of the anterior frontal space, thin development of the corpus callosum, and a slight decrease in white matter in the brain. No new skin manifestations were noted during the 1-year follow-up period.

## 3 Discussion

Fever and convulsions were the patient's primary manifestations, resulting in a misdiagnosis of central nervous system infection at the initial consultation. The child's blood count progressed from a mono- to trilineage reduction, accompanied by a decrease in fibrinogen, suggesting involvement of the blood system and coagulation function. Hypoproteinemia, aspartate aminotransferase elevation, ferritin, triglycerides, and soluble interleukin-2 receptors were markedly elevated on the premise that sepsis and bacteremia have been ruled out, causing us to consider MAS or HLH. The hemophagocytosis observed during repeated bone marrow aspiration and the hepatosplenomegaly supported our diagnosis. The CD107a excitation test revealed a defect in NK cell degranulation function. The patient's family refused further follow-up the altered NK cell function due to the effective treatment. We speculated the abnormal NK cell function was secondary to the low NK cell count. The clinical characteristics of this disease include non-relieving hyperthermia, hepatosplenomegaly, lymphadenopathy, hemorrhage, and encephalopathy. The laboratory features include pancytopenia, liver dysfunction, coagulopathy, decreased erythrocyte sedimentation rate, hypoproteinemia, hypertriglyceridemia, and methemoglobinemia. Bone marrow examination results indicated macrophages with hemophagic activity (8). MAS most commonly occurs during the active or flare-up phase of the disease (9) and may also precede or occur within a few days of the apparent manifestations of the primary disease (10).

MAS lesions can present as widespread erythematous plaques, maculopapular rashes, allergic rashes, and urticaria-like rashes all over the body (11–13), and 65% of skin lesions develop in the early stage of MAS/HLH (14). Meanwhile, the most common clinical manifestations of NLE are cutaneous lupus or heart block, alone or in combination. Furthermore, one-third of children with cutaneous NLE exhibit extracutaneous organ involvement (15). Typical skin lesions most commonly develop on the face and periorbital area, colloquially described as an “owl's eye” or “eye patch” rash, followed by the scalp, trunk, extremities, neck, and areas prone to friction, presenting as annular or round or oval erythema, papules, macules, scaly rashes, and crusted changes (16, 17).

Our observation of the periorbital “owl-eye” facial rash, as well as positive serum anti-SSA, anti-SSB, and antinuclear antibodies, led to a presumptive diagnosis of NLE. This diagnosis was further clarified after verifying the maternal origin of the antibodies after refining the ANA profile of the child's mother, who was diagnosed with Sjorgen's syndrome. The patient was finally diagnosed with NLE complicated with MAS.

Lesions are one of the important clinical features shared by NLE and MAS, and the dermatopathology of NLE includes keratinocyte damage in the epidermis and mononuclear cell infiltration in the dermis (18). The classic skin histopathology of the MAS is characterized by lymphocyte and macrophage infiltration of the dermis without epidermal abnormalities (19). Although hemophagocytosis in skin biopsy alone is insufficient to diagnose MAS, it may be a marker of MAS and a means to differentiate between NLE and MAS. Because the laboratory tests and physical examination were sufficient to diagnose MAS, we did not perform a skin biopsy.

In addition to the difference in pathological features, the prognosis of the MAS and NLE are also different. All cutaneous manifestations of MAS can completely resolve within a few days typically. In contrast, NLE lesions last an average of 15–17 weeks (17), with a small percentage of residual telangiectasia, dyspigmentation, and atrophic scarring (20). In the present case, the rash had completely resolved about 4 months after discharge.

Fever in the present case was non-sustainable, this differs from the sudden, unrelieved hyperthermic pattern (temperature greater than 39.5°C) commonly observed in MAS (21). Meanwhile, the fever was accompanied by rash, which developed in synchrony with a temperature drop, and we speculated that the fever was caused by NLE and associated with rash-associated vasculitis. During treatment, we also found that rash remission precedes the recovery of relevant biochemical indicators, suggesting that changes in the rash could be used as an indicator to judge whether the disease is being treated effectively.

Convulsions were another important clinical manifestation accompanying fever, suggesting neurological involvement. Central nervous system (CNS) involvement in NLE can be mostly asymptomatic, with radiological/ultrasound manifestations of transient diffuse reduction of white matter, basal ganglion calcification, ventricular enlargement, hydrocephalus (22), and aseptic meningitis confirmed by lumbar puncture (23). NLE with symptoms of CNS involvement primarily presents with spastic lower limb paralysis, hypospadias, myelopathy, focal epilepsy, impaired consciousness, and hydrocephalus requiring surgery (24). Unlike NLE, 35%–66% of patients with MAS have been reported to have CNS involvement, with altered mental status and seizures being the most common symptom (21, 25). Other rare manifestations include emotional changes (26), intracranial hemorrhage, and cerebral edema (27). Cortical and white matter changes have been observed on cranial MRI (28). In the present case, the child had frequent convulsions with altered mental status, and cranial MRI showed white matter changes. However, it was difficult to distinguish whether the neurologic involvement was caused by MAS alone or whether both NLE and MAS were involved. To date, it is now well established that neurologic involvement, cerebrospinal fluid abnormalities, and activated partial thromboplastin time >44.3 s are all risk factors for death in children with HLH (29–31), and the latter can be used as an independent factor to predict death. The present case had all three of these risk factors, indicating that the child was at significant risk of death. Additionally, the child had multiple organ involvement, including the skin, liver, blood, heart, brain, and lungs, including pleural effusion.

In terms of treatment, antibiotics were the main treatment regimen at the initial stage of admission, and the child's rash, fever, and convulsions were not significantly relieved.

It was not until the administration of high-dose immunoglobulins and non-shock-dose hormone therapy that effectively controlled the child's condition. Despite the many organs involved, treatment was effective, which is rare for NLE complicated by MAS. With the clearance of the maternal antibodies from the body, the child's skin, blood, and hepatobiliary abnormalities resolved, with no significant neurological sequelae observed at later follow-up.

## 4 Conclusion

We believe that high-dose immunoglobulins and non-pulsed-dose corticosteroids could be used as a first-line therapy for NLE complicated by MAS. However, whether they can be used as first-line treatment in patients with other autoimmune diseases complicated by MAS needs to be verified with more clinical data. In addition, fever can be a clinical manifestation of NLE, especially cutaneous lupus. Rash recession could be used to judge whether the disease is being effectively controlled by treatment. Eventually, we propose that for children with rash and recurrent fever, routine autoimmune antibody screening should be performed early, which may reduce unnecessary treatments such as antibiotics. Finally, it is worth noting that although we did not conduct a skin biopsy in this case report, the differentiation in histopathology between NLE and MAS prompted us to propose that a skin biopsy can aid in the early identification of both.

At present, the child has been followed up for 1.5 years, and his motor and intellectual development have shown no delays compared to that of his peers. A cranial MRI 4 months after discharge revealed that the corpus callosum was thin and the white matter was decreased. This suggests that an abnormal MRI result does not necessarily indicate abnormal development. However, long-term follow-up of children with NLE is necessary.

## Data Availability

The raw data supporting the conclusions of this article will be made available by the authors, without undue reservation.

## References

[1] Lee LA. Neonatal lupus erythematosus. J Invest Dermatol. (1993) 100:S9–13. 10.1111/1523-1747.ep12355173 8423406

[2] Izmirly PM Halushka MK Rosenberg AZ Whelton S Rais-Bahrami K Nath DS Clinical and pathologic implications of extending the spectrum of maternal autoantibodies reactive with ribonucleoproteins associated with cutaneous and now cardiac neonatal lupus from SSA/ro and SSB/la to U1RNP. Autoimmun Rev. (2017) 16:980–3. 10.1016/j.autrev.2017.07.013 28709760 PMC12035821

[3] Buyon JP Hiebert R Copel J Craft J Friedman D Katholi M Autoimmune-associated congenital heart block: demographics, mortality, morbidity and recurrence rates obtained from a national neonatal lupus registry. J Am Coll Cardiol. (1998) 31:1658–66. 10.1016/S0735-1097(98)00161-2 9626848

[4] Erden A Fanouriakis A Kiliç L Sari A Armağan B Bilgin E Geoepidemiology and clinical characteristics of neonatal lupus erythematosus: a systematic literature review of individual patients’ data. Turk J Med Sci. (2020) 50:281–90. 10.3906/sag-1910-39 31905489 PMC7164747

[5] Ravelli A. Macrophage activation syndrome. Curr Opin Rheumatol. (2002) 14:548–52. 10.1097/00002281-200209000-00012 12192253

[6] Alongi A Naddei R De Miglio L Natoli V Ravelli A. Macrophage activation syndrome in pediatrics. Pediatr Allergy Immunol. (2020) 31(Suppl 24):13–5. 10.1111/pai.13158 32017214

[7] Janka GE. Hemophagocytic syndromes. Blood Rev. (2007) 21:245–53. 10.1016/j.blre.2007.05.001 17590250

[8] Schulert GS Grom AA. Pathogenesis of macrophage activation syndrome and potential for cytokine- directed therapies. Annu Rev Med. (2015) 66:145–59. 10.1146/annurev-med-061813-012806 25386930 PMC5846123

[9] Rath T Kuo TT Baker K Qiao S-W Kobayashi K Yoshida M The immunologic functions of the neonatal fc receptor for IgG. J Clin Immunol. (2013) 33(Suppl 1):S9–17. 10.1007/s10875-012-9768-y 22948741 PMC3548031

[10] Perez MF Torres MED Buján MM Lanoël A Cervini AB Pierini AM. Neonatal lupus erythematosus: a report of four cases. An Bras Dermatol. (2011) 86:347–51. 10.1590/S0365-05962011000200021 21603821

[11] Shimozawa H Kono Y Matano M Suzuki Y Koike Y Yada Y Cytokine profile in two siblings with neonatal lupus erythematosus. Pediatr Int. (2015) 57:1211–4. 10.1111/ped.12723 26711923

[12] Heijstek V Habib M Van Der Palen R Van Doorn R Muller PH. Macrophage activation syndrome in a newborn: report of a case associated with neonatal lupus erythematosus and a summary of the literature. Pediatr Rheumatol Online J. (2021) 19:13. 10.1186/s12969-021-00500-w 33568193 PMC7877111

[13] Canna SW De Jesus AA Gouni S Brooks SR Marrero B Liu Y An activating NLRC4 inflammasome mutation causes autoinflammation with recurrent macrophage activation syndrome. Nat Genet. (2014) 46:1140–6. 10.1038/ng.3089 25217959 PMC4177369

[14] Henter JI Elinder G Söder O Ost A. Incidence in Sweden and clinical features of familial hemophagocytic lymphohistiocytosis. Acta Paediatr Scand. (1991) 80:428–35. 10.1111/j.1651-2227.1991.tb11878.x 2058392

[15] Lee LA. The clinical spectrum of neonatal lupus. Arch Dermatol Res. (2009) 301:107–10. 10.1007/s00403-008-0896-4 18797891

[16] Weston WL Morelli JG Lee LA. The clinical spectrum of anti-ro-positive cutaneous neonatal lupus erythematosus. J Am Acad Dermatol. (1999) 40:675–81. 10.1016/S0190-9622(99)70146-5 10321592

[17] Neiman AR Lee LA Weston WL Buyon JP. Cutaneous manifestations of neonatal lupus without heart block: characteristics of mothers and children enrolled in a national registry. J Pediatr. (2000) 137:674–80. 10.1067/mpd.2000.109108 11060534

[18] Lee LA Gaither KK Coulter SN Norris DA Harley JB. Pattern of cutaneous immunoglobulin G deposition in subacute cutaneous lupus erythematosus is reproduced by infusing purified anti-ro (SSA) autoantibodies into human skin-grafted mice. J Clin Invest. (1989) 83:1556–62. 10.1172/JCI114052 2651477 PMC303861

[19] Favara BE. Hemophagocytic lymphohistiocytosis: a hemophagocytic syndrome. Semin Diagn Pathol. (1992) 9:63–74. 1561489

[20] Levy R Briggs L Silverman E Pope E Lara-Corrales I. Cutaneous sequelae in neonatal lupus: a retrospective cohort study. J Am Acad Dermatol. (2020) 83:440–6. 10.1016/j.jaad.2019.09.083 31626881

[21] Stéphan JL Koné-Paut I Galambrun C Mouy R Bader-Meunier B Prieur AM. Reactive haemophagocytic syndrome in children with inflammatory disorders. A retrospective study of 24 patients. Rheumatology (Oxford). (2001) 40:1285–92. 10.1093/rheumatology/40.11.1285 11709613

[22] Prendiville JS Cabral DA Poskitt KJ Au S Sargent MA. Central nervous system involvement in neonatal lupus erythematosus. Pediatr Dermatol. (2003) 20:60–7. 10.1046/j.1525-1470.2003.03014.x 12558850

[23] Song JY Park SE Byun J-H Lee N Han YM Byun SY Neonatal lupus erythematosus as a rare cause of fever in young infants. J Clin Med. (2021) 10:3195. 10.3390/jcm10143195 34300361 PMC8306892

[24] Chen CC Lin KL Chen CL Wong AM-K Huang JL. Central nervous system manifestations of neonatal lupus: a systematic review. Lupus. (2013) 22:1484–8. 10.1177/0961203313509294 24142583

[25] Minoia F Davì S Horne A Demirkaya E Bovis F Li C Clinical features, treatment, and outcome of macrophage activation syndrome complicating systemic juvenile idiopathic arthritis: a multinational, multicenter study of 362 patients. Arthritis Rheumatol. (2014) 66:3160–9. 10.1002/art.38802 25077692

[26] Shi N Wang X Zou L Yang X Ma Q Lu M. Case report: macrophage activation syndrome and widespread neuroimaging abnormality in childhood-onset systemic lupus erythematosus. Front Pediatr. (2021) 9:767115. 10.3389/fped.2021.767115 34970517 PMC8713754

[27] Kinjo N Hamada K Hirayama C Shimizu M. Role of plasma exchange, leukocytapheresis, and plasma diafiltration in management of refractory macrophage activation syndrome. J Clin Apher. (2018) 33:117–20. 10.1002/jca.21570 28730667

[28] Lilleby V Haydon J Sanner H Krossness BK Ringstad G Flatø B. Severe macrophage activation syndrome and central nervous system involvement in juvenile dermatomyositis. Scand J Rheumatol. (2014) 43:171–3. 10.3109/03009742.2013.863968 24588361

[29] Harnchoowong S Soponkanaporn S Vilaiyuk S Lerkvaleekul B Pakakasama S. Central nervous system involvement and thrombocytopenia as predictors of mortality in children with hemophagocytic lymphohistiocytosis. Front Pediatr. (2022) 10:941318. 10.3389/fped.2022.941318 36147804 PMC9485874

[30] Horne A Trottestam H Aricò M Egeler RM Filipovich AH Gadner H Frequency and spectrum of central nervous system involvement in 193 children with haemophagocytic lymphohistiocytosis. Br J Haematol. (2008) 140:327–35. 10.1111/j.1365-2141.2007.06922.x 18076710

[31] Chen T-Y Hsu M-H Kuo H-C Sheen J-M Cheng M-C Lin Y-J. Outcome analysis of pediatric hemophagocytic lymphohistiocytosis. J Formos Med Assoc. (2021) 120:172–9. 10.1016/j.jfma.2020.03.025 32307323

